# Supplementary material for: Meiotic DNA breaks activate a streamlined phospho-signaling response that largely avoids protein-level changes
Source: Life Sci Alliance. 2022 Sep 1;5(10):e202201454. doi: 10.26508/lsa.202201454 (PMC9438802; doi:10.26508/lsa.202201454)
Supplement: Supplementary file 1 [file LSA-2022-01454_TableS1.docx]

**Supplemental Table 1: DNA break-dependent phosphorylation events**

| **Protein** | **Site** | **Approach for categorization** |
| --- | --- | --- |
| ABD1 | 12 | presence/absence |
| ABF1 | 189 | fold enrichment |
| ABF1 | 193 | fold enrichment |
| ABF1 | 467 | fold enrichment |
| AHP1 | 2 | presence/absence |
| AIM21 | 85 | fold enrichment |
| AKL1 | 12 | presence/absence |
| APC1 | 310 | presence/absence |
| APN1 | 346 | presence/absence |
| ASF1 | 270 | fold enrichment |
| ASF1 | 265 | presence/absence |
| ASG1 | 166 | presence/absence |
| ATG13 | 429 | presence/absence |
| ATG13 | 649 | presence/absence |
| AZF1 | 312 | presence/absence |
| AZF1 | 313 | presence/absence |
| AZF1 | 325 | presence/absence |
| BBC1 | 895 | fold enrichment |
| BBC1 | 894 | fold enrichment |
| BDF1 | 630 | fold enrichment |
| BDF1 | 626 | presence/absence |
| BIR1 | 747 | fold enrichment |
| BIR1 | 381 | fold enrichment |
| BIR1 | 751 | fold enrichment |
| BOI1 | 655 | presence/absence |
| BUB1 | 384 | presence/absence |
| BUD14 | 640 | presence/absence |
| BUD14 | 641 | presence/absence |
| BUD14 | 642 | presence/absence |
| BUD21 | 137 | fold enrichment |
| BYE1 | 210 | fold enrichment |
| CBF1 | 45 | fold enrichment |
| CBF1 | 48 | fold enrichment |
| CDC4 | 53 | presence/absence |
| CHS3 | 538 | fold enrichment |
| CIN5 | 196 | fold enrichment |
| CIN5 | 85 | presence/absence |
| CIN5 | 89 | presence/absence |
| CIN8 | 259 | fold enrichment |
| CIN8 | 261 | fold enrichment |
| CIT2 | 21 | presence/absence |
| CKB2 | 12 | presence/absence |
| CMR1 | 224 | fold enrichment |
| CMR1 | 79 | fold enrichment |
| CUS1 | 104 | presence/absence |
| CYR1 | 241 | presence/absence |
| DAL81 | 17 | fold enrichment |
| DBF4 | 84 | fold enrichment |
| DBF4 | 473 | fold enrichment |
| DBF4 | 496 | presence/absence |
| DBF4 | 501 | presence/absence |
| DBP7 | 56 | presence/absence |
| DCP2 | 686 | fold enrichment |
| DDC1 | 558 | presence/absence |
| DGK1 | 3 | presence/absence |
| DIF1 | 102 | presence/absence |
| DIF1 | 104 | presence/absence |
| DMA2 | 206 | presence/absence |
| DPB4 | 183 | fold enrichment |
| DPS1 | 301 | presence/absence |
| DST1 | 115 | fold enrichment |
| DUN1 | 10 | presence/absence |
| ECM11 | 150 | fold enrichment |
| ELC1 | 2 | presence/absence |
| ENT1 | 160 | fold enrichment |
| ENT1 | 163 | fold enrichment |
| ENT1 | 392 | presence/absence |
| ENT2 | 468 | presence/absence |
| ENT2 | 470 | presence/absence |
| ESL1 | 174 | presence/absence |
| EXO1 | 663 | presence/absence |
| EXO1 | 664 | presence/absence |
| FBP1 | 13 | fold enrichment |
| FIN1 | 68 | fold enrichment |
| GAL11 | 789 | presence/absence |
| GCD1 | 296 | presence/absence |
| GCD14 | 302 | fold enrichment |
| GCD14 | 299 | presence/absence |
| GFA1 | 332 | presence/absence |
| GIP2 | 117 | presence/absence |
| GIP2 | 120 | presence/absence |
| GLC7 | 3 | fold enrichment |
| GPA2 | 23 | presence/absence |
| GTT1 | 56 | fold enrichment |
| HAS1 | 12 | fold enrichment |
| HAS1 | 14 | presence/absence |
| HBT1 | 1034 | fold enrichment |
| HBT1 | 1036 | fold enrichment |
| HBT1 | 965 | fold enrichment |
| HED1 | 40 | presence/absence |
| HED1 | 42 | presence/absence |
| HHT1 | 58 | presence/absence |
| HOG1 | 176 | fold enrichment |
| HOP1 | 22 | presence/absence |
| HPC2 | 307 | fold enrichment |
| HPC2 | 388 | presence/absence |
| HRB1 | 27 | fold enrichment |
| HRR25 | 438 | presence/absence |
| HRT1 | 15 | presence/absence |
| HSP12 | 21 | fold enrichment |
| HSP12 | 24 | fold enrichment |
| HSP12 | 59 | fold enrichment |
| HSP26 | 208 | presence/absence |
| HSP26 | 211 | presence/absence |
| HTA1 | 129 | fold enrichment |
| HXT2 | 17 | fold enrichment |
| IGD1 | 64 | fold enrichment |
| IGD1 | 83 | presence/absence |
| IGO2 | 119 | fold enrichment |
| INO1 | 2 | presence/absence |
| IOC2 | 629 | presence/absence |
| IPL1 | 5 | fold enrichment |
| IRR1 | 14 | fold enrichment |
| IRR1 | 12 | fold enrichment |
| IRR1 | 21 | presence/absence |
| IVY1 | 32 | presence/absence |
| LEO1 | 339 | presence/absence |
| MAF1 | 179 | presence/absence |
| MAK21 | 2 | presence/absence |
| MBP1 | 133 | fold enrichment |
| MCM10 | 66 | presence/absence |
| MCM4 | 52 | fold enrichment |
| MDS3 | 621 | fold enrichment |
| MEC3 | 368 | fold enrichment |
| MEC3 | 369 | fold enrichment |
| MEK1 | 486 | presence/absence |
| MEK1 | 356 | presence/absence |
| MET12 | 120 | presence/absence |
| MLH1 | 441 | fold enrichment |
| MMS4 | 2 | fold enrichment |
| MON1 | 130 | fold enrichment |
| MOT1 | 679 | presence/absence |
| MRC1 | 121 | presence/absence |
| MRE11 | 558 | fold enrichment |
| MSC3 | 57 | fold enrichment |
| MSC3 | 64 | fold enrichment |
| MSH6 | 102 | fold enrichment |
| MTL1 | 481 | fold enrichment |
| MTR4 | 84 | fold enrichment |
| NAB2 | 2 | presence/absence |
| NET1 | 1025 | fold enrichment |
| NET1 | 1026 | fold enrichment |
| NET1 | 1024 | fold enrichment |
| NET1 | 440 | presence/absence |
| NET1 | 439 | presence/absence |
| NGG1 | 231 | presence/absence |
| NNK1 | 689 | fold enrichment |
| NOG2 | 60 | fold enrichment |
| NOT3 | 446 | presence/absence |
| NPL3 | 224 | fold enrichment |
| NUP53 | 95 | fold enrichment |
| NUP60 | 10 | fold enrichment |
| NUP60 | 458 | fold enrichment |
| NUP60 | 460 | fold enrichment |
| NUP60 | 483 | presence/absence |
| NUR1 | 441 | presence/absence |
| OLA1 | 67 | presence/absence |
| PAP1 | 550 | fold enrichment |
| PCT1 | 67 | fold enrichment |
| PEX3 | 118 | fold enrichment |
| PEX3 | 119 | fold enrichment |
| PFK1 | 3 | fold enrichment |
| PIL1 | 45 | fold enrichment |
| PRI2 | 16 | fold enrichment |
| PRI2 | 17 | fold enrichment |
| PUP2 | 128 | fold enrichment |
| RAD1 | 40 | fold enrichment |
| RAD14 | 360 | fold enrichment |
| RAD16 | 109 | presence/absence |
| RAD17 | 350 | presence/absence |
| RAD23 | 121 | presence/absence |
| RAD24 | 637 | fold enrichment |
| RAD26 | 27 | presence/absence |
| RAD52 | 205 | presence/absence |
| RAD54 | 132 | presence/absence |
| RAD7 | 64 | fold enrichment |
| RCK2 | 187 | presence/absence |
| RDI1 | 9 | presence/absence |
| REC114 | 55 | fold enrichment |
| RED1 | 483 | fold enrichment |
| RED1 | 484 | fold enrichment |
| RED1 | 486 | fold enrichment |
| RED1 | 473 | fold enrichment |
| RED1 | 517 | fold enrichment |
| RED1 | 518 | fold enrichment |
| RFA2 | 115 | presence/absence |
| RFM1 | 83 | presence/absence |
| RFX1 | 226 | presence/absence |
| RFX1 | 173 | presence/absence |
| RGT1 | 284 | fold enrichment |
| RIF1 | 1362 | fold enrichment |
| RIM15 | 555 | presence/absence |
| RPC53 | 234 | presence/absence |
| RPS16A | 15 | fold enrichment |
| RPS2 | 30 | fold enrichment |
| RPS7B | 14 | presence/absence |
| RRB1 | 5 | fold enrichment |
| RRM3 | 125 | presence/absence |
| RRP12 | 1050 | presence/absence |
| RRP36 | 41 | fold enrichment |
| RRP36 | 42 | fold enrichment |
| RSC9 | 44 | presence/absence |
| RTF1 | 17 | presence/absence |
| RTF1 | 2 | presence/absence |
| RTG3 | 241 | fold enrichment |
| RTG3 | 246 | fold enrichment |
| RTG3 | 269 | presence/absence |
| RTG3 | 236 | presence/absence |
| RTS2 | 160 | presence/absence |
| RTT107 | 591 | fold enrichment |
| RTT107 | 593 | fold enrichment |
| RTT107 | 735 | fold enrichment |
| RTT107 | 800 | fold enrichment |
| RTT107 | 806 | fold enrichment |
| RTT107 | 255 | presence/absence |
| SEC16 | 2139 | presence/absence |
| SEC16 | 2141 | presence/absence |
| SEC3 | 254 | presence/absence |
| SEC3 | 256 | presence/absence |
| SEF1 | 273 | presence/absence |
| SEG1 | 48 | presence/absence |
| SFT2 | 2 | presence/absence |
| SGF73 | 19 | fold enrichment |
| SGF73 | 22 | presence/absence |
| SGO1 | 421 | fold enrichment |
| SGO1 | 423 | fold enrichment |
| SGO1 | 426 | fold enrichment |
| SGS1 | 482 | presence/absence |
| SGS1 | 606 | presence/absence |
| SGV1 | 417 | fold enrichment |
| SHP1 | 226 | presence/absence |
| SIR2 | 23 | presence/absence |
| SIR3 | 263 | presence/absence |
| SIR4 | 692 | fold enrichment |
| SIR4 | 342 | presence/absence |
| SKI7 | 88 | fold enrichment |
| SKI7 | 90 | fold enrichment |
| SLD2 | 150 | fold enrichment |
| SLD2 | 151 | fold enrichment |
| SLI15 | 97 | presence/absence |
| SLK19 | 216 | fold enrichment |
| SLM1 | 157 | fold enrichment |
| SLM1 | 158 | fold enrichment |
| SMB1 | 67 | fold enrichment |
| SPC105 | 144 | presence/absence |
| SPC110 | 60 | fold enrichment |
| SPC24 | 2 | presence/absence |
| SPC29 | 230 | fold enrichment |
| SPC29 | 231 | fold enrichment |
| SPC29 | 248 | fold enrichment |
| SPC29 | 249 | fold enrichment |
| SPC29 | 250 | fold enrichment |
| SPG4 | 93 | fold enrichment |
| SPO13 | 139 | fold enrichment |
| SPO13 | 134 | presence/absence |
| SPP1 | 18 | fold enrichment |
| SPP41 | 563 | fold enrichment |
| SPP41 | 564 | fold enrichment |
| SPT7 | 78 | fold enrichment |
| SRP40 | 394 | presence/absence |
| SSD1 | 489 | presence/absence |
| SSN2 | 748 | presence/absence |
| SSN2 | 521 | presence/absence |
| STB3 | 337 | fold enrichment |
| STB3 | 2 | presence/absence |
| STE20 | 192 | presence/absence |
| STE20 | 195 | presence/absence |
| STU1 | 1000 | fold enrichment |
| SUB2 | 2 | fold enrichment |
| SUB2 | 12 | fold enrichment |
| SUB2 | 13 | fold enrichment |
| SUM1 | 722 | fold enrichment |
| SUM1 | 858 | fold enrichment |
| SUM1 | 859 | fold enrichment |
| SWI3 | 88 | fold enrichment |
| SWI3 | 185 | fold enrichment |
| SYH1 | 687 | fold enrichment |
| TAF3 | 345 | fold enrichment |
| TAF3 | 346 | fold enrichment |
| TAF8 | 212 | fold enrichment |
| TAF8 | 215 | fold enrichment |
| TCB3 | 1340 | fold enrichment |
| THO1 | 72 | fold enrichment |
| THO1 | 68 | presence/absence |
| THS1 | 2 | presence/absence |
| TIF4632 | 913 | fold enrichment |
| TIF5 | 184 | presence/absence |
| TOA2 | 102 | presence/absence |
| TOA2 | 95 | presence/absence |
| TOP1 | 24 | fold enrichment |
| TPO3 | 52 | presence/absence |
| TRM10 | 16 | presence/absence |
| TSL1 | 157 | fold enrichment |
| UBC5 | 12 | fold enrichment |
| UBP1 | 776 | fold enrichment |
| UBP13 | 463 | fold enrichment |
| UGX2 | 186 | fold enrichment |
| ULP1 | 264 | fold enrichment |
| ULP1 | 48 | fold enrichment |
| ULP2 | 773 | fold enrichment |
| ULP2 | 929 | fold enrichment |
| ULP2 | 936 | fold enrichment |
| ULP2 | 937 | fold enrichment |
| ULS1 | 204 | fold enrichment |
| UNG1 | 23 | presence/absence |
| USV1 | 369 | presence/absence |
| UTP22 | 10 | fold enrichment |
| UTP5 | 629 | presence/absence |
| UTP5 | 637 | presence/absence |
| VTC2 | 187 | presence/absence |
| XRN1 | 1510 | fold enrichment |
| XRS2 | 783 | presence/absence |
| YBR285W | 117 | presence/absence |
| YCS4 | 495 | presence/absence |
| YDL199C | 61 | fold enrichment |
| YDR090C | 306 | presence/absence |
| YDR090C | 309 | presence/absence |
| YDR239C | 74 | presence/absence |
| YDR239C | 681 | presence/absence |
| YER079W | 10 | presence/absence |
| YER079W | 9 | presence/absence |
| YET1 | 172 | presence/absence |
| YGR130C | 803 | fold enrichment |
| YGR130C | 44 | presence/absence |
| YHR097C | 69 | fold enrichment |
| YJL206C | 90 | presence/absence |
| YLR257W | 238 | presence/absence |
| YPQ2 | 136 | presence/absence |
| ZIP1 | 549 | presence/absence |
| ZIP1 | 75 | presence/absence |
| ZIP1 | 546 | presence/absence |
| ZIP1 | 865 | presence/absence |
