# Supplementary material for: Meiotic DNA breaks activate a streamlined phospho-signaling response that largely avoids protein-level changes
Source: Life Sci Alliance. 2022 Sep 1;5(10):e202201454. doi: 10.26508/lsa.202201454 (PMC9438802; doi:10.26508/lsa.202201454)
Supplement: Supplementary file 2 [file LSA-2022-01454_TableS2.docx]

**Supplemental Table 2: Phosphosites enriched in *spo11-YF***

| **Protein** | **Site** | **Approach for categorization** |
| --- | --- | --- |
| ABF1 | 159 | presence/absence |
| ACF4 | 9 | presence/absence |
| CRZ1 | 453 | presence/absence |
| CUS1 | 114 | presence/absence |
| DNA2 | 17 | fold enrichment |
| DPS1 | 30 | presence/absence |
| IMH1 | 25 | presence/absence |
| KEL1 | 1019 | presence/absence |
| KKQ8 | 113 | presence/absence |
| LIF1 | 261 | presence/absence |
| MLF3 | 145 | presence/absence |
| PDS1 | 212 | presence/absence |
| PDS1 | 213 | presence/absence |
| RPL24A;RPL24B | 83;83 | presence/absence |
| RPL24A;RPL24B | 86;86 | presence/absence |
| SEC3 | 301 | presence/absence |
| THO1 | 48 | presence/absence |
| UFD1 | 314 | presence/absence |
| VPS27 | 280 | presence/absence |
| YFR016C | 49 | presence/absence |
| YJL163C | 59 | presence/absence |
| YJL163C | 61 | presence/absence |
| YOL036W | 547 | presence/absence |
| YPL150W | 900 | presence/absence |
| ARG82 | 98 | fold enrichment |
| CKI1 | 54 | fold enrichment |
| GET2 | 60 | fold enrichment |
| PIN4 | 638 | fold enrichment |
| ARO80 | 96 | fold enrichment |
| DNA2 | 236 | fold enrichment |
| DNA2 | 237 | fold enrichment |
| ESC2 | 71 | fold enrichment |
| ESC2 | 73 | fold enrichment |
| ESC2 | 76 | fold enrichment |
| PIB2 | 68 | fold enrichment |
| PIB2 | 70 | fold enrichment |
| REC104 | 110 | fold enrichment |
| RHO4 | 54 | fold enrichment |
| RHO4 | 55 | fold enrichment |
| RTT107 | 720 | fold enrichment |
| STU1 | 1113 | fold enrichment |
| UBP1 | 652 | fold enrichment |
| XRS2 | 349 | fold enrichment |
| AVO1 | 144 | presence/absence |
| BNR1 | 621 | presence/absence |
| BUL1 | 195 | presence/absence |
| DRE2 | 212 | presence/absence |
| EDE1 | 1062 | presence/absence |
| FAS2 | 1440 | presence/absence |
| GYP6 | 436 | presence/absence |
| HOP1 | 553 | presence/absence |
| MCM10 | 18 | presence/absence |
| MCM10 | 68 | presence/absence |
| OSH3 | 447 | presence/absence |
| OSH3 | 449 | presence/absence |
| OSH3 | 452 | presence/absence |
| PKH1 | 303 | presence/absence |
| PXA2 | 776 | presence/absence |
| RAD7 | 118 | presence/absence |
| REC107 | 37 | presence/absence |
| RGA2 | 733 | presence/absence |
| RGC1 | 1033 | presence/absence |
| RHO4 | 58 | presence/absence |
| RRP9 | 76 | presence/absence |
| SEC16 | 1633 | presence/absence |
| SEC16 | 1634 | presence/absence |
| SEC16 | 1640 | presence/absence |
| SGS1 | 643 | presence/absence |
| SGT1 | 166 | presence/absence |
| SKG3 | 814 | presence/absence |
| SUI2 | 52 | presence/absence |
| SVL3 | 551 | presence/absence |
| SWI3 | 230 | presence/absence |
| SWI3 | 231 | presence/absence |
| SWI3 | 91 | presence/absence |
| TSL1 | 137 | presence/absence |
| VHS3 | 264 | presence/absence |
| VID27 | 417 | presence/absence |
| YAT2 | 848 | presence/absence |
| YEL043W | 895 | presence/absence |
| YPL150W | 807 | presence/absence |
