# Supplementary material for: Meiotic DNA breaks activate a streamlined phospho-signaling response that largely avoids protein-level changes
Source: Life Sci Alliance. 2022 Sep 1;5(10):e202201454. doi: 10.26508/lsa.202201454 (PMC9438802; doi:10.26508/lsa.202201454)
Supplement: Supplementary file 3 [file LSA-2022-01454_TableS3.docx]

**Supplemental Table 3: Genotypes of the strains used in this study**

| **Strain** | **Genotype** |
| --- | --- |
| H10355 | *MATa, ho::LYS2, lys2, HIS, URA, spo11-Y135F-HA-URA3, pph3Δ::LEU2, ndt80Δ::TRP1 MATalpha, ho::LYS2, lys2, HIS, URA , TRP1, spo11-Y135F-HA-URA3, pph3Δ::LEU2, ndt80Δ::TRP1* |
| H10423 | *MATalpha, ho::LYS2, lys2, HIS, URA, LEU2, pph3Δ::LEU2, ndt80Δ::TRP1 MATa, ho::LYS2, lys2, HIS, URA, LEU, ndt80Δ::TRP1, pph3Δ::LEU2* |
| H11604 | *MATa, ho::LYS2, lys2, ura3, leu2::hisG, HIS, trp1::hisG, spo11-Y135F-HA-URA3, pph3Δ::LEU2, ndt80Δ::TRP1, FRD1-13myc::HIS3MX6 MATalpha, ho::LYS2, lys2, ura3, leu2::hisG, HIS?, trp1::hisG, spo11-Y135F-HA-URA3, pph3Δ::LEU2, ndt80Δ::TRP1, FRD1-13myc::HIS3MX6* |
| H11605 | *MATa, ho::LYS2, lys2, ura3, leu2::hisG, HIS, trp1::hisG, spo11-Y135F-HA-URA3, pph3Δ::LEU2, ndt80Δ::TRP1, FRD1-13myc::HIS3MX6 MATalpha, ho::LYS2, lys2, ura3, leu2::hisG, HIS, trp1::hisG, pph3Δ::LEU2, ndt80Δ::TRP1, FRD1-13myc::HIS3MX6.* |
| H11641 | *MATa, ho::LYS2, lys2, ura3, leu2::hisG, his3::hisG, trp1::hisG,  DBP2-13myc::HIS3MX6, pph3Δ::LEU2, ndt80Δ::TRP1, spo11-Y135F-HA-URA3 MATalpha, ho::LYS2, lys2, ura3, leu2::hisG, his3::hisG, trp1::hisG,  DBP2-13myc::HIS3MX6, pph3Δ::LEU2, ndt80Δ::TRP1* |
| H11642 | *MATa, ho::LYS2, lys2, ura3, leu2::hisG, his3::hisG, trp1::hisG,  DBP2-13myc::HIS3MX6, pph3Δ::LEU2, ndt80Δ::TRP1, spo11-Y135F-HA-URA3 MATalpha, ho::LYS2, lys2, ura3, leu2::hisG, his3::hisG, his4X, trp1::hisG,  DBP2-13myc::HIS3MX6, pph3Δ::LEU2, ndt80Δ::TRP1, spo11-Y135F-HA-URA3* |
| H11746 | *MATalpha, ho::LYS2, lys2, ura3, leu2::hisG, his3::hisG, trp1::hisG, pph3Δ::LEU2, spo11-Y135F-HA-URA3, ndt80Δ::TRP1, RNR4-13myc::HIS3MX6 MATa, ho::LYS2, lys2, ura3, leu2::hisG, his3::hisG, trp1::hisG, pph3Δ::LEU2, spo11-Y135F-HA-URA3, ndt80Δ::TRP1, RNR4-13myc::HIS3MX6* |
| H11747 | *MATalpha, ho::LYS2, lys2, ura3, leu2::hisG, his3::hisG, trp1::hisG, pph3Δ::LEU2,ndt80Δ::TRP1, RNR4-13myc::HIS3MX6 MATa, ho::LYS2, lys2, ura3, leu2::hisG, his3::hisG, trp1::hisG, pph3Δ::LEU2, spo11-Y135F-HA-URA3, ndt80Δ::TRP1, RNR4-13myc::HIS3MX6* |
| H119 | *MATa, ho::LYS2, lys2, ura3, leu2::hisG,  MATalpha, ho::LYS2, lys2, ura3, leu2::hisG,  his4B::LEU2, arg4-Bgl II his4X::LEU2 (Bam)-URA3, arg4-Nsp* |
| H10963 | *MATa, ho::LYS2, lys2, ura3, leu2::hisG, his3::hisG, trp1::hisG, hrr25-2a MATalpha, ho::LYS2, lys2, URA3, LEU2, his3::hisG, trp1::hisG, hrr25-2a* (*2a: Serine438Alanine,Threonine453Alanine*) |
| H11031 | *MATa, ho::LYS2, lys2, ura3, leu2::hisG, his3::hisG, trp1::hisG leu2::pURA3-TetR-GFP::LEU2, HRR25-13MYC::HIS3MX6 MATalpha, ho::LYS2, lys2, ura3, leu2::hisG, his3::hisG, trp1::hisG leu2::pURA3-TetR-GFP::LEU2, ura3::TETOx224::URA3, HRR25-13MYC::HIS3MX6* |
| H7797 | *MATa, ho::LYS2, lys2, ura3, leu2::hisG, his3::hisG, trp1::hisG MATalpha, ho::LYS2, lys2, URA3, LEU2, HIS3, TRP1* |
| H11368 | *MATa, ho::LYS2, ura3, leu2::hisG, his3::hisG, trp1::hisG,  MATalpha, ho::LYS2, lys2, leu2::hisG, HIS, URA3, TRP1,  pph3Δ::LEU2 pph3Δ::LEU2* |
| H11505 | *MATa, ho::LYS2, lys2, URA3, LEU2, his3::hisG, trp1::hisG MATalpha, ho::LYS2, lys2, ura3, leu2::hisG, HIS3, TRP1 pCLB2-DBP2::KanMX6 pCLB2-DBP2::KanMX6* |
| H11506 | *MATa, ho::LYS2, lys2, leu2::hisG, HIS3, TRP1, ura3,  MATalpha, ho::LYS2, lys2, leu2::hisG, his3::hisG, trp1::hisG, URA3,  pph3Δ::LEU2, pCLB2-DBP2::KanMX6 pph3Δ::LEU2, pCLB2-DBP2::KanMX6* |
