# Supplementary material for: Meiotic DNA breaks activate a streamlined phospho-signaling response that largely avoids protein-level changes
Source: Life Sci Alliance. 2022 Sep 1;5(10):e202201454. doi: 10.26508/lsa.202201454 (PMC9438802; doi:10.26508/lsa.202201454)
Supplement: Supplementary file 4 [file LSA-2022-01454_TableS4.docx]

**Supplemental Table 4: m/z windows used for DIA**

| **Start** | **End** | **Width** |
| --- | --- | --- |
| 350 | 385 | 35 |
| 384 | 412 | 28 |
| 411 | 434 | 23 |
| 613 | 636 | 23 |
| 454 | 474 | 20 |
| 493 | 513 | 20 |
| 552 | 572 | 20 |
| 473 | 494 | 21 |
| 512 | 533 | 21 |
| 532 | 553 | 21 |
| 433 | 455 | 22 |
| 571 | 593 | 22 |
| 592 | 614 | 22 |
| 635 | 659 | 24 |
| 658 | 683 | 25 |
| 682 | 709 | 27 |
| 708 | 738 | 30 |
| 737 | 771 | 34 |
| 770 | 808 | 38 |
| 807 | 848 | 41 |
| 847 | 899 | 52 |
| 898 | 966 | 68 |
| 965 | 1,068 | 103 |
| 1,067 | 1,650 | 583 |
